# Analysis of PK11195 concentrations in rodent whole blood and tissue samples by rapid and reproducible chromatographic method to support target-occupancy PET studies

**DOI:** 10.1016/j.jchromb.2019.04.026

**Published:** 2019-06-15

**Authors:** Agnė Stadulytė, Carlos José Alcaide-Corral, Tashfeen Walton, Christophe Lucatelli, Adriana Alexandre S. Tavares

**Affiliations:** a University/BHF Centre for Cardiovascular Science, University of Edinburgh, UK; b Edinburgh Preclinical Imaging (EPI), University of Edinburgh, UK; c Edinburgh Imaging, Queen's Medical Research Institute, University of Edinburgh, UK

**Keywords:** PET, positron emission tomography, PK/PD, pharmacokinetics/pharmacodynamics, HPLC, high performance liquid chromatography, TO, target occupancy, TSPO, translocator 18 kDa protein, Positron emission tomography, PK/PD, HPLC, PK11195, Target occupancy

## Abstract

In Positron Emission Tomography (PET) research, it is important to assess not only pharmacokinetics of a radiotracer *in vivo*, but also of the drugs used in blocking/displacement PET studies. Typically, pharmacokinetic/pharmacodynamic (PK/PD) analyses of drugs used in rodent PET studies are based on population average pharmacokinetic profiles of the drugs due to limited blood volume withdrawal while simultaneously maintaining physiological homeostasis. This likely results in bias of PET data quantification, including unknown bias of target occupancy (TO) measurements. This study aimed to develop a High Performance Liquid Chromatography (HPLC) method for PK/PD quantification of drugs used in preclinical rodent PET research, specifically the translocator 18 kDa protein (TSPO) selective drug, PK11195, that used sub-millilitre blood volumes. The lowest detection limit for the proposed HPLC method ranged between 7.5 and 10 ng/mL depending on the method used to calculate the limit of detection, and the measured average relative standard deviation for intermediate precision was equal to 17.2%. Most importantly, we were able to demonstrate a significant difference between calculated PK11195 concentrations at 0.5, 1, 2, 3, 5, 15 and 30 min post-administration and individually measured whole blood levels (significance level range from *p* < 0.05 to *p* < 0.001; one-way ANOVA, Dunnet's post hoc test, p < 0.05). The HPLC method developed here uses sub-millilitre sample volumes to reproducibly assess PK/PD of PK11195 in rodent blood. This study highlights the importance of individually measured PK/PD drug concentrations when quantifying the TO from blocking/displacement rodent PET experiments.

## Highlights

• A new HPLC method to measure low drug levels in blood and tissue was developed.
• This method uses sub-millilitre samples to reproducibly assess PK/PD of drugs.
• Population-based drug level curves overestimated real drug plasma levels.
• New HPLC method can be rapidly applied to rodent PET target-occupancy studies.

## 1 Introduction

Positron Emission Tomography (PET) is a molecular imaging modality with numerous applications in both preclinical and clinical research environment. This non-invasive technique offers great sensitivity allowing for *in vivo* imaging of biochemical processes by injecting a radioactive isotope-labelled compound at tracer levels (typically picomolar to micromolar range) [1]. One of the key applications of PET imaging is to support target-occupancy (TO) studies with newly developed drugs, i.e. to serve as a companion biomarker. Furthermore, TO studies can be pivotal at confirming selective target engagement of novel PET radiotracers [2]. In human clinical trials, the percentage TO quantification is supported by pharmacokinetic/pharmacodynamic (PK/PD) analysis of arterial or venous blood for each individual in order to determine plasma or whole blood levels of the blocking/displacement agent at a given time point [3,4]. Also, this approach has been extensively and directly applied to PET TO studies in large animals [5,6]. Unfortunately, individual PK/PD analysis of drugs used in PET TO studies of small animals, specifically, rodents, is less standard [7,8] and at best the impact of using population-based assessments in rodents is poorly described. Accurate quantification of PK/PD on an individual basis is rationally justified because technical errors during drug administration (e.g. faulty injection or partial volume injected), as well as individual variability in metabolism (e.g. due to either genetic or environmental factors [9]) cannot be adequately corrected if a population average PK/PD curve is used. Overcoming this bias would benefit accuracy of the preclinical PET TO image quantification and would facilitate confident selection of novel radiotracers and drugs suitable for further translational research.

Foreseeably, the main limiting factors for implementing individual PK/PD analysis as standard practice in small animal PET studies are: (1) the extremely low levels of blocking or displacement agents concentration in the whole blood/plasma which might represent challenges in terms of detection limits of chromatographic instrumentation, such as high performance liquid chromatography (HPLC); and (2) most critically, the small blood sampling volume allowed for small animals [10]. Here we propose a rapid and practical analytical HPLC method which allows for detection of blood and tissue concentrations of a well-known selective 18 kDa translocator protein (TSPO) drug, PK11195 (1-[2-chlorophenyl]-N-[1-methyl-propyl]-3-isoquinoline carboxamide), in small (sub-mililitre) volumes of rat whole blood and tissue samples. Whole blood analysis was preferred over plasma analysis, as PK11195 is known to bind to erythrocytes and plasma proteins [11,12].

## 2 Materials and methods

### 2.1 Reagents and solvents

PK11195 (>99% purity) was purchased from Abcam (Cambridge, UK). The gradient grade HiperSolv CHROMANORM® acetonitrile (>99.9% purity) was acquired from VWR Chemicals (Lutterworth, UK). The ultrapure water was obtained by using Millipore Smart2Pure water purification system. Prior to use, both acetonitrile and water were filtered through 0.22 μm Magna nylon membranes (GVS, USA) using a chemical duty vacuum pump (Millipore, US). DMSO was purchased from Fisher Bioreagents (US).

### 2.2 Animals and sample collection

All experiments were authorized by the local University of Edinburgh animal welfare and ethical review committee and were conducted in accordance with the Home Office Animals (Scientific Procedures) Act 1986. Healthy naïve adult male Sprague-Dawley rats (373 ± 20 g, mean ± SD, *n* = 6) were anaesthetised using 2.0–2.5% isoflurane (50/50 oxygen/nitrous oxide, 1 L/min). The femoral artery was cannulated for blood collection post drug administration as described previously [13] and PK11195 (5 mg/kg in 100% DMSO, 100 μL) was injected via tail vein (bolus i.v.). Animals have been divided into two groups: animals 1, 2 and 3 had their arterial blood samples taken at 0.5, 1, 2 and 3 min post-PK11195 injection; whereas animals 4, 5 and 6 had their arterial blood samples taken at 5, 15, 30 and 60 min post-PK11195 injection. In total, 4 mL of blood have been collected into heparinized tubes (50 IU/mL) from each animal, i.e. 1 mL per time point, in order to respect the blood volume collection limits for rats [10]. Immediately after, each 1 mL sample was sub-aliquot into 300 μL samples for subsequent HPLC analysis. The tissue samples, including heart, lungs, brain and spleen were collected at a terminal point, i.e. 3 min (animals 1, 2, 3) and 60 min (animals 4, 5, 6) post-PK11195 injection. Following collection, both whole blood and tissue samples were frozen on dry ice and stored at −80 °C until processed. Analysis of whole-blood samples rather than plasma processed samples is common place to drugs that bind to blood cells [14,15].

### 2.3 Analytical HPLC method development

#### 2.3.1 HPLC system and PK11195 detection

HPLC analysis was performed on a Dionex Ultimate 3000 UHPLC (Thermo Scientific, UK). The chromatographic separation was carried out at 25 °C using a Luna® C18(2) 150 mm × 4.6 mm, 10 μm column (Phenomenex, UK). The mobile phase consisted of 70% acetonitrile in water. A reference standard PK11195 sample has been prepared at 1 mg/mL concentration in acetonitrile:water (70:30). A 100 μL sample of the PK11195 reference standard was injected and the column was eluted with an isocratic mobile phase (1 mL/min flow for 10 min). The peak of PK11195 in the samples was detected by UV/Vis detector at 331 nm.

#### 2.3.2 Chromatographic method validation

In order to test the validity of the analytical HPLC method, the HPLC method repeatability and intermediate precision, linearity, limit of detection (LOD) and limit of quantitation (LOQ) were calculated.

In order to determine the linearity of the method, the calibration curve of PK11195 was generated by injecting freshly prepared PK11195 dissolved in acetonitrile:water (70:30) (concentration range 7.5–1000 ng/mL, *n* = 3 per concentration) using the analytical HPLC method described in Section 2.3.1. The concentration was plotted versus the average peak area. The two lowest concentrations at which the PK11195 peak could be visually identified (7.5 and 25 ng/mL) and two concentrations in between (10 and 15 ng/mL) were selected for analysis of system repeatability and injected onto the HPLC system 6 consecutive times using the same conditions. The experiment was repeated on another day for intermediate precision testing. Measuring system repeatability and intermediate precision at concentrations around the LOD/LOQ values provides additional confidence on the robustness of the obtained measures, as the analysis of the samples around these working limits are more challenging and represent worst-case conservative scenario. Both repeatability and intermediate precision were expressed as percentage relative standard deviation (%RSD). The LOD and LOQ for the proposed HPLC method were calculated as previously described [16] and using the formulae:

(1) $$\mathrm{LOD}=3.3\times \frac{\sigma }{S}$$

(2) $$\mathrm{LOQ}=10\times \frac{\sigma }{S}$$

where, *σ* is the standard deviation of the y-intercept, and *S* is the slope of the calibration curve. Additionally, LOD was determined by calculating signal to noise ratio and peak detectability was defined as a S/N ratio >2, according to the International Council for Harmonisation (ICH) standards [17].

### 2.4 PK11195 PK/PD measurements in rat blood and tissue

#### 2.4.1 Blood and tissue sample preparation and HPLC analysis

On the day of the HPLC experiment, ultrapure water was added to the defrosted tissue samples (heart, lungs and spleen 1:1, w/v; and brain 1:3, w/v) in order to facilitate homogenization. The homogenized tissues and arterial blood samples were centrifuged at 2000 rpm 4 °C (24 position rotor, Heraeus Megafuge 8R, Thermo Scientific) for 5 min. The whole blood and tissue supernatants were collected and centrifugation was repeated 3 times under the same conditions in order to recover as much whole blood and tissue supernatant as possible. Freezing-thawing cycle of the whole blood sample causes cell lysis, therefore the resultant supernatant represents the whole blood drug content, in line with previously described blood sample collection and storage/processing methods [18,19]. The cell-free tissue and whole blood and tissue supernatants were denatured using acetonitrile (1:1.4, v/v) and centrifuged at 2000 rpm 4 °C for 4 min. Following protein precipitation, the final supernatants were collected for HPLC analysis.

The prepared samples were analysed using the method described in Section 2.3.1. The measured PK11195 peak area was converted to concentration by applying linear regression equation obtained from the calibration curve.

#### 2.4.2 Comparison of calculated versus measured PK11195 concentration in rat whole blood

It was important to assess how accurate the calculated PK11195 concentrations were relative to measured PK11195 concentration using the HPLC method described in this study. The results obtained from the analysed blood samples collected at 0.5, 1, 2, 3, 5, 15, 30 and 60 min after the administration of PK11195 were compared to the concentration of PK11195 estimated from the two-phase exponential regression equation (Eq. 3) derived from the population-based blood PK/PD curve.

### 2.5 Statistical analysis and PK/PD data fitting

Statistical analysis of the results was performed using GraphPad Prism 5 (GraphPad Software, USA). Statistical significance was estimated by one-way ANOVA followed by Bonferroni or Dunnett's post-hoc tests. One-phase decay exponential regression model was applied to determine PK/PD of PK11195 in individual animals, and two-phase decay exponential regression model was applied in order to estimate the population pharmacokinetic whole blood profile of PK11195.

## 3 Results

### 3.1 Analytical HPLC method development

**Fig. 1:**
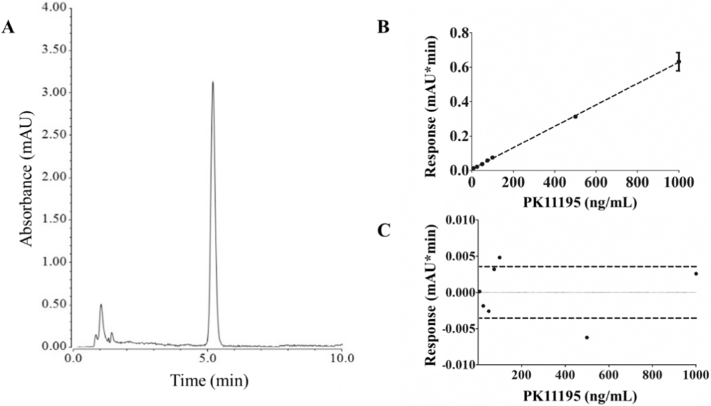
Linearity of the proposed analytical HPLC method. A) Representative chromatogram of PK11195 1000 ng/mL (*t*_*R*_ = 5.19 min). B) Calibration curve of PK11195 (concentration range 7.5–1000 ng/mL; *n* = 3 per concentration, ±95% CI shown as data points error bars). Linear equation: Y = 0.0006× + 0.0073, where Y = response (mAU·min), x = PK11195 concentration (ng/mL), *r*^*2*^ = 0.999. C) Residuals plot of the calibration curve and 95% CI shown as dashed lines. CI = confidence interval.

The average PK11195 retention time (*t*R) was 5.18 ± 0.04 min (mean ± SD, *n* = 80 different injections used for method development, Fig. 1A). Collected data showed the HPLC signal measured with the developed method was linear relative to the sample concentration (*r*^*2*^ = 0.999, Fig. 1B) and the fitting residuals were ± 0.005 mAU·min (Fig. 1C) with only two outliers outside the 95% confidence interval correspondent to concentrations 100 ng/mL and 500 ng/mL. The measured method repeatability on day 1 and 2 was 11.3% and 9.5% on average, respectively. The mean value for the intermediate precision was 17.3% (Table 1).

**Table 1:** Measured HPLC peak area for different PK11195 concentrations, repeatability and reproducibility of the developed analytical method. All data shown as average response±SD (*n* = 6), D1 = the 1st day of sample analysis and D2 = the 2nd day of sample analysis.

	Concentration of PK11195			
	7.5 ng/mL	10 ng/mL	15 ng/mL	25 ng/mL
Average response D1 (mAU·min)	0.010 ± 0.002	0.011 ± 0.001	0.012 ± 0.002	0.020 ± 0.003
Repeatability D1 (%)	22	11	7	5
Average response D2 (mAU·min)	0.009 ± 0.001	0.010 ± 0.001	0.013 ± 0.001	0.023 ± 0.001
Repeatability D2 (%)	14	13	5	6
Intermediate precision D1 vs. D2 (%)	24	13	15	17

The calculated LOD for the present analytical HPLC method was 11.7 ng/mL, and LOQ was equal to 35.4 ng/mL when determined using standard equations (Eq. (1), (2)). The S/N ratio analysis showed this ratio was higher than 2 for all the PK11195 concentrations tested: 2.4 ± 0.6 for 7.5 ng/mL, 3.0 ± 0.5 for 10 ng/mL, 3.1 ± 0.4 for 15 ng/mL and 4.5 ± 0.5 for 25 ng/mL (mean ± SD, *n* = 12).

### 3.2 PK11195 PK/PD measurements in rat blood and tissue

In order to test the feasibility and reproducibility of the proposed analytical HPLC method with sub-millilitre injection volumes of biological samples containing PK11195, upon collection, each 1 mL whole blood sample was divided into three equal volumes (i.e. 3 x ~300 μL), frozen for storage, then defrosted, centrifuged and denatured to obtain supernatant and analysed using HPLC. Each one out of three 300 μL whole blood samples was processed and analysed on a different day during the course of three days. On average, the inter-sample variability was 15.5% (Table 2), in line with inter-day variability assessed using reference standard solutions of PK11195. The retention time of PK11195 in whole blood and tissue samples remained consistent throughout the study (5.18 ± 0.03 min, *n* = 94) and it was identical to the retention time of the reference standard.

**Table 2:** Inter-sample PK/PD variability of PK11195 in biological samples. Average PK11195 whole blood concentration ± SD at different time points post-PK11195 injection for individual animals (*n* = 2 for 5 min whole blood samples from animals 5 and 6, and *n* = 3 for all remaining time points per animal) were measured using the proposed analytical HPLC method. The level of significance in the sample group (i.e. PK11195 concentration at particular time point across three animals) was determined using one-way ANOVA followed by Bonferroni's post hoc test (*p* < 0.05).

Time, min	PK11195 (ng/mL)	RSD, %	PK11195 (ng/mL)	RSD, %	PK11195 (ng/mL)	RSD, %	*p* value
	*Animal 1*		*Animal 2*		*Animal 3*		
0.5	895 ± 47	5.3	702 ± 135	19.2	467 ± 29	6.1	0.0067
1	762 ± 49	6.5	388 ± 70	18	395 ± 39	9.8	0.0007
2	682 ± 64	9.3	357 ± 50	13.8	327 ± 36	11.1	0.0008
3	627 ± 42	6.7	313 ± 23	7.3	292 ± 5	1.7	<0.0001
	*Animal 4*		*Animal 5*		*Animal 6*		
5	93 ± 47	50.4	488;415	–	240; 215	–	0.0034
15	73 ± 33	45.4	259 ± 19	7.3	138 ± 7	5.4	0.0005
30	48 ± 3	5.9	144 ± 16	11.1	94 ± 7	7.6	0.0003
60	35 ± 24	69.6	52 ± 10	18.5	48 ± 11	22.6	0.5791

**Fig. 2:**
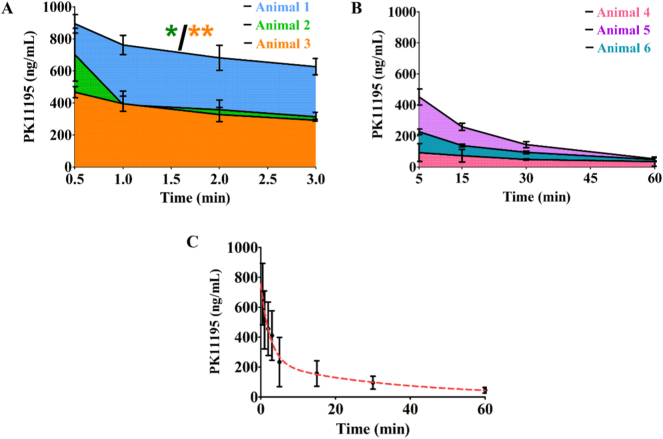
Individual and population-based PK/PD curves of PK11195 in rat whole blood. A) One-phase exponential regression plot representing the concentration of PK11195 in the whole blood at 0.5, 1, 2, 3 min post-intravenous administration of the animals 1, 2, 3 and B) 5, 15, 30 and 60 min post-injection of the animals 4, 5, 6 (mean ± SD, *n* *=* *3* per time point). C) Measured whole blood PK11195 concentrations at 0.5, 1, 2, 3, 5, 15, 30 and 60 min post-injection in the animals 1 to 6, represented by black dots and error bars (mean ± SD, *n* *=* *3* per time point), and population-based two-phase exponential regression plot (red dotted line) representing the calculated concentration of PK11195 in the whole blood over time. This regression plot was used to derive Eq. 3. Statistically significant differences were determined using ANOVA, Bonferroni Post-hoc test, *p* < 0.05(*), *p* < 0.01(**). (For interpretation of the references to colour in this figure legend, the reader is referred to the web version of this article.)

Fig. 2 presents the PK/PD response of PK11195 in rat whole blood over time. Measured half-life (*T*_*1/2*_) for animal 1, 2 and 3 were 0.6 min, 0.2 min and 0.8 min, respectively. Area under the curve (AUC) for animals 1, 2 and 3 were 1790, 980.8 and 886.1 ng/mL·min, respectively (Fig. 2A). Measured *T*_*1/2*_ for animals 4, 5 and 6 were 15.9 min, 11.9 min and 12.2 min, respectively. Area under the curve (AUC) for animals 4, 5 and 6 were 2989, 9516 and 5709 ng/mL·min, respectively (Fig. 2B). By combining the data obtained from all individual animals in the study and by applying two-phase exponential regression model to this data set, a population average curve was obtained (Fig. 2C) and an equation was derived, which can be used to determine PK11195 concentration in rat whole blood at any given time point:

(3) $$\left[\mathrm{PK}11195\right]=7.8+523.2\times e^{-0.4\mathrm{time}}+222.4\times e^{-0.03\mathrm{time}}$$

**Fig. 3:**
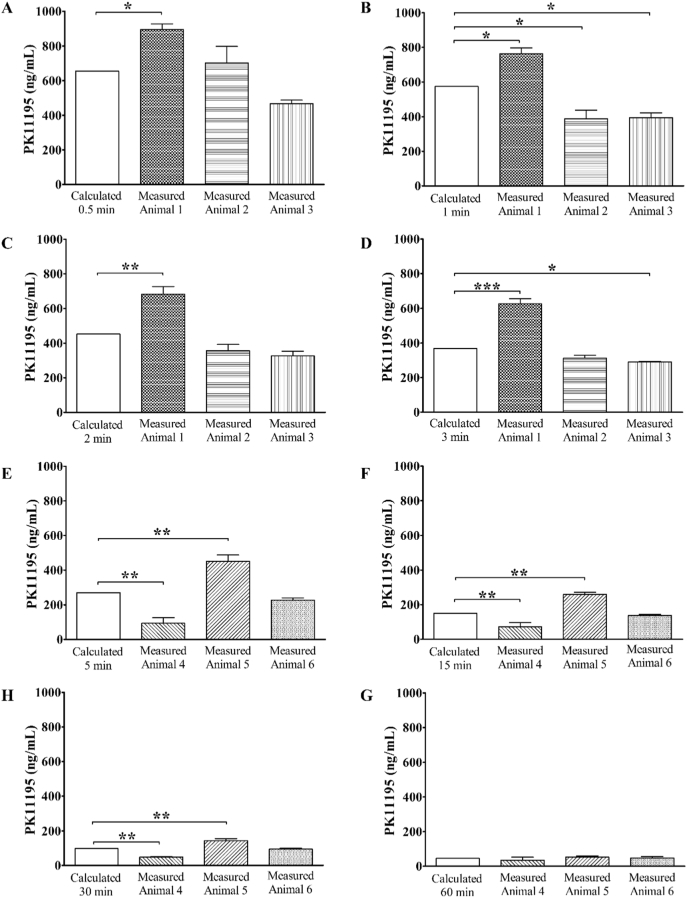
Comparison of calculated versus measured concentration of PK11195 in whole blood. Calculated vs measured PK11195 concentration in rodent whole blood at 0.5 min (A), 1 min (B), 2 min (C), and 3 min (D) post-injection of PK11195 (mean ± SD, *n* = 3, one-way ANOVA followed by Dunnetts' multiple comparison test (*p* < 0.05)). Calculated vs measured PK11195 concentration in rodent blood at 5 min (E), 15 min (F), 30 min (G), and 60 min (H) post-injection of PK11195 (mean ± SD, *n* = 3, one-way ANOVA followed by Dunnetts' multiple comparison test (*p* < 0.05)). p < 0.05(*), p < 0.01(**), *p* < 0.001(***).

The calculated PK11195 concentration from the population average curve was compared with the measured PK11195 concentration in rat whole blood at 0.5, 1, 2, 3, 5, 15, 30 and 60 min post-injection (Fig. 3). The results showed that the PK11195 concentration obtained using Eq. 3 was significantly different from measured PK11195 concentration in at least one animal at every time point, with the exception of samples collected at 60 min post-injection, (one-way ANOVA followed by Dunnetts' multiple comparison test, *p* < 0.05).

**Fig. 4:**
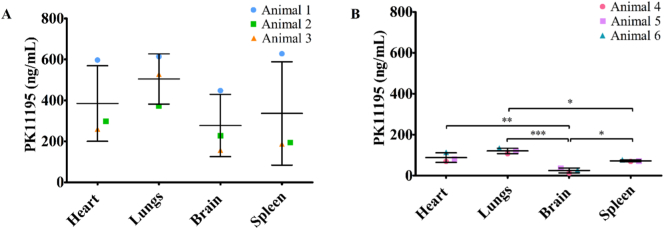
Concentration of PK11195 in the different tissues. The concentration of PK11195 across different organs at A) 3 min and B) 60 min post-injection. Results presented as mean ± SD, *n* = 3. Statistically significant differences were determined using one-way ANOVA followed by Bonferroni multiple comparison test, *p* < 0.05(*), *p* < 0.01(**), *p* < 0.001(***).

The developed HPLC method was also applied to investigate concentration of PK11195 in tissue. The PK/PD analysis showed that PK11195 was actively and rapidly cleared from the blood and kinetically transferred into the organs (Fig. 4). The average PK11195 concentration was not significantly different at 3 min post-injection across all the organs tested in the study (Fig. 3A), however 1 h post-injection the drug levels in the brain were significantly lower when compared to the heart, lungs and spleen as well as lower in the spleen when compared to the lungs (*p* = 0.0003; one-way ANOVA, Bonferroni multiple comparison test).

## 4 Discussion

In this study, an analytical HPLC method was developed for the detection of nanomolar concentrations of the TSPO selective drug, PK11195, in small size biological samples. The developed method has shown to be feasible, reproducible and linear. Robustness of the method was successfully challenged with nanomolar concentrations and sub-millilitre volumes. Results presented here highlight and quantify the bias associated with population-based PK/PD analysis versus individual analysis of small animal data, with consequent impact on drug studies, namely PET TO data quantification. Even though the described HPLC method is valid only for PK11195, upon developing appropriate HPLC methods, the same approach could be potentially applied for testing PK/PD of different drugs.

The proposed analytical HPLC method for PK11195 detection proved to be feasible using both chemical and biological samples (whole blood and tissue). On average, the method repeatability was 17.3% for chemical PK11195 dilutions and 13.5% for whole blood samples containing tracer amounts of PK11195. A 5 to 15% repeatability limit in chromatographic measurements has been defined for analysis at trace levels [20], and even though the average intermediate precision among PK11195 measurements in chemical samples was higher than this limit, the value for PK11195 detection in blood samples was within the required range (Table 1, Table 2). Other validation criteria were met as the method showed exceptionally good linearity even at LOD, which was at nanomolar range. Our results are in line with previously published data indicating that, in chromatographic analysis, smaller values of the detection limit and the quantification limit were better described by visual analysis or S/N ratio, albeit larger variations [21]. Furthermore, we have quantified the S/N ratio for the peaks observed in the HPLC trace at the four lowest concentrations, as an additional line of evidence to test the sensitivity of the method. That analysis has shown the LOD was 7.5 ng/mL with a S/N ratio of 2.4 ± 0.6 (mean ± SD, *n* = 12). Taken together, all this criteria pinpoint the developed HPLC method as a reproducible and sensitive analytical tool for PK11195 levels above 7.5 ng/mL.

Previously described methods for HPLC analysis of PK11195 were overall similar to the method proposed in this study, but there was a large range of HPLC mobile phases and UV wavelengths used for PK11195 elution and detection. The UV light wavelengths ranged from 254 nm to 310 nm, and organic solvent:water ratio of the mobile phase ranged between 30:70 to 80:20 [[22], [23], [24]]. Using our chromatographic system, and after testing a wide range of UV wavelengths, we concluded that the optimal wavelength to detect PK11195 in biological samples was 331 nm (data not shown). In 2008, Wala et al. [25] has also described a HPLC method of PK11195 detection and PK/PD analysis in rats. They have reported PK11195 detection at 240 nm UV channel using K₂HPO₄:methanol:acetonitrile (35:63:2) as mobile phase and Supelco LC-8 column with a retention time twice as long as the one obtained with our method (*tR* = 10 min versus *tR* = 5.18 min). Even though %RSD for repeatability and reproducibility at 500 ng/mL PK11195 were 7.7% and 7.9%, respectively, it is worth noting that the concentration used by the authors was 50–65 fold higher than the concentrations used for repeatability and intermediate precision testing using our method, hence more favourable %RSD values. Despite these differences, the LOD was 10 ng/mL. Therefore, the results reported in the current study are comparable with the findings of Wala et al. in spite of the different methods used for blood processing. This is likely due to the known reversible PK11195 binding to erythrocytes and plasma proteins – albumin and α1-acid glycoprotein [11,12]. As the PK11195 in plasma binds to tissues, the erythrocyte-bound and plasma protein-bound PK11195 may dissociate, causing fluctuations in plasma PK11195 levels. As a result, equilibrium between free and bound PK11195 levels is never reached. This justifies the use of whole blood drug for the HPLC analysis, as ultimately PK11195 present in whole blood contributes to the extent of its effects. Furthermore, other previously reported HPLC studies of drugs that are known to bind to the red blood cells have also used whole blood measurements and reported a blood collection and storage method similar the one described in this manuscript [18,19].

Most importantly, we were also able to demonstrate that the usage of population average PK/PD data of PK11195 for rodent PET TO image quantification can result in notable quantitative bias (0.3 to 1.7 fold difference between calculated and measured PK11195 whole blood levels). The larger inter-individual variability is likely to be a result of operational artefacts and individual variability in metabolism in different animals. Still, this issue can be overcome by applying the proposed analytical HPLC method as standard PK/PD determination test, in order to improve the accuracy of the preclinical PET TO data quantification and accelerate confident translation of novel radiotracers and drugs from the preclinical to clinical research. PET TO small animal studies play an important role in the early phase drug development, as they assist dosing regimen optimisation for subsequent clinical trials [[26], [27], [28]]. Therefore, the adherence to individual-based PK/PD analysis, such as the one reported here, would significantly improve the accuracy of preclinical PET TO quantification, and consequently supports the translational process.

Even though there are other slightly more sensitive chromatographic systems for PK11195 detection available, such as LC/MS [[29], [30], [31]], our proposed method proved to be relatively inexpensive, rapid and reliable. The adherence to this individual approach could be rapidly realised across laboratories. Another advantage of the developed method is that the small blood sample size of approximately 300 μL is sufficient for the robust analysis. It offers the opportunity for reliable quantitative PK/PD analysis of PK11195 in smaller species.

In addition to whole blood data, in this study we have also demonstrated that it is possible to detect PK11195 in rodent tissues, such as heart, lungs, brain and spleen, using the proposed sample processing and analytical HPLC methods. The data becomes extremely useful when determining a direct relationship between blocking drug concentration and percentage occupancy in the target tissue during preclinical PET radiotracer development process and early preclinical phases of drug discovery. Even though in the clinical drug development environment there is no readily available access to tissue [32], given the ability of the proposed HPLC method to perform well even with small quantities (sub-millilitre) blood samples, this approach could potentially be applied for small human tissue samples obtained during tissue biopsy procedures.

## 5 Conclusions

A robust chromatographic method to measure PK11195 concentration in small volume rodent whole blood samples and tissue was developed. This method uses sub-millilitre sample volumes to rapidly and reproducibly assess PK/PD of PK11195 in small animals. Data collected highlighted the need for individually measured PK/PD drug concentration levels in samples, in order to derive more accurate drug exposure levels compared with population-based estimates often used in preclinical research with small animals. Even though different HPLC methods would have to be developed for different drugs, this proof-of-concept study using PK11195 suggests that the proposed methodological approach is feasible even with sub-millilitre volumes and, if applied more widely, has potential to help eliminate the bias in small animal TO PET data quantification.
